# Genome Analysis of *Vallitalea guaymasensis* Strain L81 Isolated from a Deep-Sea Hydrothermal Vent System

**DOI:** 10.3390/microorganisms6030063

**Published:** 2018-07-04

**Authors:** Anders Schouw, Francesca Vulcano, Irene Roalkvam, William Peter Hocking, Eoghan Reeves, Runar Stokke, Gunhild Bødtker, Ida Helene Steen

**Affiliations:** 1 Department of Biological Sciences and KG Jebsen Centre for Deep Sea Research, University of Bergen, N-5020 Bergen, Norway; anders.schouw@uib.no (A.S.); F.vulcano@uib.no (F.V.); irene.roalkvam@uib.no (I.R.); William.hocking@metis.no (W.P.H.); Runar.stokke@uib.no (R.S.); 2 Department of Earth Science and KG Jebsen Centre for Deep Sea Research, University of Bergen, N-5020 Bergen, Norway; Eoghan.reeves@uib.no; 3 Centre for Integrated Petroleum Research (CIPR), Uni Research AS, Nygårdsgaten 112, N-5008 Bergen, Norway; Gunhild.bodtker@uni.no

**Keywords:** *Vallitalea guaymasensis*, hydrothermal vent, syntrophy, whole-genome sequence

## Abstract

*Abyssivirga alkaniphila* strain L81^T^, recently isolated from a black smoker biofilm at the Loki’s Castle hydrothermal vent field, was previously described as a mesophilic, obligately anaerobic heterotroph able to ferment carbohydrates, peptides, and aliphatic hydrocarbons. The strain was classified as a new genus within the family *Lachnospiraceae*. Herein, its genome is analyzed and *A. alkaniphila* is reassigned to the genus *Vallitalea* as a new strain of *V. guaymasensis*, designated *V. guaymasensis* strain L81. The 6.4 Mbp genome contained 5651 protein encoding genes, whereof 4043 were given a functional prediction. Pathways for fermentation of mono-saccharides, di-saccharides, peptides, and amino acids were identified whereas a complete pathway for the fermentation of *n*-alkanes was not found. Growth on carbohydrates and proteinous compounds supported methane production in co-cultures with *Methanoplanus limicola*. Multiple confurcating hydrogen-producing hydrogenases, a putative bifurcating electron-transferring flavoprotein—butyryl-CoA dehydrogenase complex, and a Rnf-complex form a basis for the observed hydrogen-production and a putative reverse electron-transport in *V. guaymasensis* strain L81. Combined with the observation that *n*-alkanes did not support growth in co-cultures with *M. limicola*, it seemed more plausible that the previously observed degradation patterns of crude-oil in strain L81 are explained by unspecific activation and may represent a detoxification mechanism, representing an interesting ecological function. Genes encoding a capacity for polyketide synthesis, prophages, and resistance to antibiotics shows interactions with the co-occurring microorganisms. This study enlightens the function of the fermentative microorganisms from hydrothermal vents systems and adds valuable information on the bioprospecting potential emerging in deep-sea hydrothermal systems.

## 1. Introduction

In hydrothermal vent systems water-rock reactions produce microbial nutrients such as H_2_S, CH_4_ and H_2_, which create the basis for chemosynthetic food-webs and allow hot spots for biological activity to form in the deep ocean. This primary production results in a steady supply of organic matter that may be utilized by heterotrophic microorganisms [1,2,3]. Recently, the mesophilic heterotrophic bacterium *Abyssivirga alkaniphila* L81^T^ (=DSM 29592T = JCM 30920T) was isolated from a biofilm growing on a hydrothermal chimney at Loki’s Castle hydrothermal vent field (LCVF) [4]. The LCVF is located on the Arctic Mid-Ocean Ridge (AMOR) at 73°30′ N and 8° E, where the Mohns Ridge migrates into the Knipovich Ridge, at a depth of 2400 m [5,6]. The field is discharging black smoker fluids of 310–320 °C from four chimneys, located at two mounds, roughly 150 m apart [5,6,7]. Low temperature venting occurs at the eastern flank of the mound, where a field of small barite chimneys is found [5,8]. The high-temperature vent fluids have high concentrations of CH_4_, H_2_, and CO_2_, with CH_4_ values of 15.5 mmol kg^−1^, among the highest reported for a bare-rock hosted field [5]. The vent fluids are further characterized by a pH of 5.5, end-member H_2_S content up to 4.7 mmol kg^−1^, and very high NH_4_ concentrations. The high values for CH_4_ combined with the NH_4_ values, points to the influence of hydrothermal alteration of buried sedimentary organic matter. C_1_ to C_4_ hydrocarbons of thermogenic origin have been detected in the venting fluids [7]. The microbial mats growing on the black smokers in LCVF are dominated by chemolitoautotrophic *Epsilonproteobacteria* [3,9] supporting growth of heterotrophic *Bacteroidetes* [10,11].

Recently, an objection to our newly described genus *Abyssivirga* was proposed based on 16S rRNA phylogeny by Postec and coworkers (2017) [12]. They argued that *A. alkaniphila* should be reassigned to the genus *Vallitalea*, possibly representing a novel species, *Vallitalea alkaniphila*, if demonstrated by significant DNA-DNA hybridization and phenotyphic difference [12]. The genus *Vallitalea* belongs to the family *Defluviitaleaceae*, within *Clostridiales*, and comprises Gram negative, motile, non-spore-forming, mesophilic rods with fermentative metabolism [13,14]. So far, all strains of this genus have been isolated from marine hydrothermal systems. The type strain *V. guaymasensis* Ra1766G^T^ was isolated from microbial mats situated on sediments at Guaymas Basin [14], an area associated with hydrothermal activity and hydrocarbon seeps [15], whereas *V. pronyensis* FatNI3^T^ was isolated from a chimney in the hydrothermal alkaline springs at Prony Bay [13]. Here, we present a comparison of *A. alkaniphila* with *V. guaymasensis* RA1766G1^T^ and *V. pronyensis* FatNI3^T^ establishing *A. alkaniphila* L81^T^ as a new strain of *V. guaymasensis*, named *V. guaymasensis* L81. Moreover, a draft genome sequence of *V. guaymasensis* L81 is analyzed. Altogether, an interesting model system for the investigation of fermentative cooperation from hydrothermal vents systems has been established, along with new genomic input for bioprospecting potential attractive enzymes and new antimicrobial compounds.

## 2. Materials and Methods

Growth experiments were performed on Met II medium as described by Schouw and co-workers (2016) [4]. Cultures were grown in 30 mL glass vials, sealed with butyl rubber stoppers containing 15 mL of medium, and 15 mL of N_2_:CO_2_ (80:20) gas phase. Acetate, arabinose, butyrate, cellobiose, cellulose, chitin, decane, dextrin, formate, fructose, galactose, glucose, heptane, lactose, maltose, mannitol, mannose, octane, palatinose, pectin, pentane, peptone, propionate, pyruvate, rhamnose, ribose, starch, sucrose, tryptone, yeast extract, xylan, and xylose were tested as growth substrates. Decane, heptane, octane, pentane, pentone, and yeast extract were tested as growth substrates for co-cultures of *V. guaymasensis* and *M. limicola*.

Acetate, arabinose, butyrate, cellobiose, dextrin, formate, fructose, galactose, glucose, lactose, maltose, mannitol, mannose, palatinose, propionate, pyruvate, rhamnose, ribose, sucrose, xylan, and xylose were added to a final concentration of 20 mM. Cellulose and chitin were added to a final concentration of 0.25% *w*/*w*, and peptone, yeast extract, pectin, starch, and tryptone to a final concentration of 0.1% *w*/*w*. Decane, heptane, octane, pentane, and pentone were added to a final concentration of 1 µL/mL medium.

The production of H_2_ and CH_4_ in culture headspaces was quantified using a SRI 8610C gas chromatograph (GC), equipped with a packed column (molecular sieve 5 Å) and serially connected thermal conductivity (TCD) and flame-ionization detectors (FID), for H_2_/CH_4_ and CH_4_ detection, respectively. Nitrogen was used as a carrier gas, with a column flow rate of 20 mL/min (70 °C isothermal) and detector temperatures of 250 °C. The GC was calibrated using injected moles of commercial gas standard mixtures.

For each measurement, 0.5 mL subsamples of culture headspace gas were taken with Hamilton gas-tight syringes previously flushed with nitrogen to avoid air contamination of the serum vials. The gas samples were then transferred into a 5 mL loop filled with nitrogen, and after dilution and homogenization were then injected onto the GC column through a valve.

Measured moles of each gas were normalized to the total headspace volume. Although partitioning of gases between headspace and liquid phases is occurring, a consideration of the Henry’s Law constants for H_2_ and CH_4_ (7.8 × 10^−6^ mol/m^3^ Pa and 1.4 × 10^−5^ mol/m^3^ Pa at standard temperature in water [16]), indicates that >90% of these gases is present in the headspace at equilibrium, thus direct measurement from headspace gas subsamples accounts for the near totality of H_2_ and CH_4_ produced. Analytical uncertainty is estimated to be ±5% (2 s) for both CH_4_ and H_2_.

Interaction between microbial cells was analyzed with Fluorescence In-Situ Hybridization, as described by Glöckner and co-workers (1996) [17]. From each culture, 500 µL were fixed in 2% formaldehyde at room temperature for two hours. The cells were collected on a 0.2 µm polycarbonate filter, which was then washed twice with excess 1X PBS, air-dried and stained with fluorescently labeled oligonucleotides. *V. guaymasensis* L81 cells were targeted with EUB338 probe (5′-GCT GCC TCC CGT AGG AGT-3′) [18] labeled with Alexa488 fluorochrome, while methanogens were targeted with ARCH917 probe (5′-GTG CTC CCC CGC CAA TTC-3′) [19] labeled with Cy3 fluorochrome. For double hybridization, 5 µL of each probe (50 ng/µL stock concentration) were added to 100 µL of hybridization buffer and approximately 20 µL of the resulting mixture were applied to each filter. The hybridization step was performed in 35% formamide.

Stained filters were mounted with Immersol 518F (Carl Zeiss AG, Oberkochen, Germany) and visualized with Zeiss Axio Imager Z1 microscope (Carl Zeiss Microscopy GmbH, Göttingen, Germany), equipped with filter 38 (Alexa488) and 43 (Cy3).

For Scanning Electron Microscopy (SEM), 100 µL aliquots of each culture were fixed with 2% glutaraldehyde, at room temperature for one hour. Each aliquot was increased to 1 mL final volume using 1X PBS and was applied to a 0.2 µm polycarbonate filter to collect cells. Filters were then dehydrated with serial ethanol washes (50%, 75%, 3 × 100%), air-dried, mounted on an aluminum specimen stud with carbon tape and coated with iridium using a Gatan 682 coater (Gatan Inc., Pleasanton, CA, USA). Microbial cells were visualized by a Zeiss Supra 55VP field emission scanning electron microscope (FE-SEM; Carl Zeiss, Stockholm, Sweden), equipped with a Thermo Noran System SIX energy dispersive spectrometer (EDS) system (Carl Zeiss AS, Oslo, Norway) and an in-lens detector.

The cell wall structures in the *Vallitalea* strains were studied in detail using transmission electron microscopy (TEM). Cell cultures were embedded in LR White medium grade resin (Electron Microscopy Sciences, Pasadena, CA, USA) and later cut to 60 nm slices using a ultramikrotom (Reichert-Jung Ultracut. Leica microsystems GmbH, Wetzlar, Germany). Samples were stained with Reynold’s lead citrate solution and examined in a Jeol 1011 TEM (Jeol Ltd., Tokyo, Japan). 

DNA-DNA hybridization (DDH) included *A. alkaniphila* L81^T^, *V. guaymasensis* Ra1766G1^T^ (DSM-24848) and *V. pronyensis* FatNI3^T^ (DSM-25904). All strains were cultivated on Met II medium added 0.1% glucose and 0.05% sucrose. Cells were harvested using centrifugation at 10,000× *g* for 30 min at 4 °C. At Deutsche Sammlung von Microorganismen und Zellkulturen (DSMZ), the cells were disrupted by Constant Systems TS 0.75 KW (IUL Instruments, Barcelona, Spain), and DNA in the crude lysate was purified by chromatography on hydroxyapatite as described by Cashion and co-workers (1977) [20]. DNA-DNA hybridization was carried out as described by De Ley and co-workers (1970) [21], with modifications described by Huss and co-workers (1983) [22], using a model Cary 100 Bio UV/VIS-spectrophotometer equipped with a Peltier-thermostatted 6 × 6 multicell changer and a temperature controller with in situ temperature probe (Varian, Palo Alto, CA, USA).

Cultures grown to mid-exponential or stationary phase were fixed to glass slides and Gram-stained with crystal violet and safranin to investigate possible differences in staining characteristics. Cell wall properties was also investigated by the string test as described by Ryu (1938) [23], where 0.3% KOH was added smear from a cell pellet. Living cultures on microscopy slides were stained for flagella visualization, using the staining solution by Ryu (1937) [24] and method similar to Heimbrook and co-workers (1989) [25].

Genomic DNA for sequencing was extracted from a culture grown on Met II medium added glucose to a final concentration of 2 mM [4]. DNA was extracted by the modified Marmur method [26]. Genome sequencing was performed at the Norwegian Sequencing Centre (www.sequencing.uio.no). A 10 kb library was prepared using Pacific Bioscience 10 kb library preparation protocol and BluePippin (Sage Science, Beverly, MA, USA) for the final size selection. In total, four SMRT cells were used for sequencing the library on a Pacific Bioscience RS II Instrument (Pacific Bioscience, Menlo Park, CA, USA) in combination with the P4-C2 chemistry. The raw reads were filtered prior de novo assembly and polishing using HGAP v3, SMRT Analysis v2.2.0 (Pacific Bioscience, Menlo Park, CA, USA). The genome was annotated using RAST [27,28,29], IMG-ER [30,31,32] dbCAN [33] and eggNOG 4.5 [34]. Polyketid biosynthetic clusters were identified with antiSMASH 3.0 [35]. Putative hydrogenses annotated by RAST and IMG-ER were classified using HydDB [36]. 

This Whole Genome Shotgun project has been deposited at DDBJ/ENA/GenBank under the accession number QMDO00000000. The version described in this paper is version QMDO01000000. The annotated genome is available in the JGI GOLD under Project id: Ga0082380, and in RAST under id number: 6666666.108580.

The raw data have been deposited in the NCBI Sequence Read Archive under Study: PRJNA450742 (SRP151092), Sample: L81 genome (SRS3447209), Experiment: VguL81_WGA (SRX4282565) RUN: cell4_Vguaymasensis_s1_p0.2.bax.h5 (SRR7410928), RUN: cell3_Vguaymasensis_s1_p0.2.bax.h5 (SRR7411504), RUN: cell2_Vguaymasensis_s1_p0.2.bax.h5 (SRR7411505), RUN: cell1_Vguaymasensis_s1_p0.2.bax.h5 (SRR7411506).

## 3. Results

### 3.1. Reclassification of Abyssivirga alkaniphila L81^T^

Strain L81 was originally classified as *Abyssivirga alkaniphila* L81^T^ (=DSM 29592T = JCM 30920T) [4]. Later Postec and co-workers (2016) suggested that the 16S rRNA gene sequences of *A. alkaniphila* L81^T^ and *Valitalia guaymasensis* RA1766G1^T^, were so similar that they likely belonged to the same species [12]. To clarify this, a DNA-DNA hybridization was performed of *A. alkaniphila* L81^T^ with *V. guaymasensis* RA1766G1^T^ and *V. pronyensis*, respectively. The results revealed a similarity between *A. alkaniphila* L81^T^ and *V. guaymasensis* of 69.7% and with *V. pronyensis* of 13.35%. With the inherent variability of the method, this places *A. alkaniphila* L81^T^ and *V. guaymasensis* RA1766G1^T^ within the 70% DDH similarity recommended as the species cut-off value [37,38], supporting that they are strains of the same species. Based on a polyphasic analysis, including 16S rRNA gene similarities; DNA-DNA hybridization values; optimal temperature for growth; gram staining; flagella type and utilization of substrates, we argue that strain L81 (formerly named *Abyssivirga alkaniphila* L81^T^) should be reclassified to *Vallitalea guaymasensis* L81. Supporting data are presented in supplementary data (Supplementary Table S1). 

### 3.2. Emended Description of Vallitalea guaymasensis

Cells are gram positive, motile rods of 0.5 µm × 2–5 µm during exponential growth. Cells are usually single, but can occur in long chains. Stationary phase cells are non-motile with a spherical morphology. Growth is not observed below 15 °C or above 42 °C, and the optimal growth temperature is 37 °C. A minimum of 0.5% NaCl is required for growth, and maximum concentration of 6% NaCl is tolerated. Optimum growth rate occurs at 3% NaCl. The pH range was broad, with an optimum at pH 7.0–8.2. Reduced growth medium is required. Arabinose, cellobiose, dextrin, fructose, galactose, glucose, lactose, maltose, mannose, palatinose, pectin, peptone, ribose, starch, sucrose, tryptone, yeast extract, xylan, and xylose are utilized. Acetate, cellulose, chitin, formate, mannitol, propionate, pyruvate and rhamnose are not. A small amount of yeast extract is required for growth for some substrates, while acetate inhibit growth. Major fermentation products are H_2_ and CO_2_. Main whole-cell sugar is ribose, and trace amounts of galactose. The G + C content of chromosomal DNA is 31.7 mol%.

The type strain is *Vallitalea guaymasensis* RA1766G1^T^ (=DSM 24848 = JCM 17997).

### 3.3. Emended Description of Vallitalea Gen

Cells are motile, mesophilic rods with a fermentative and obligately organoheterotrophic metabolism. Genus is Gram-variable (Gram staining reaction) and includes both spore forming and non-spore forming strains. A minimum of 0.5% NaCl is required for growth. No quinones are detected. The major fatty acids are anteiso-C15:0, iso-C15:0, anteiso-DMA-C15:0 and C16:0.

### 3.4. Syntrophic Growth

Cultures supplemented with pentane, heptane, octane or decane, respectively, produced the same amount of methane as the negative control cultures in pure Met II medium. This indicated that the added alkanes did not support growth. When the cultures were given additional yeast extract after 11 months incubation, an immediate increase in methane-production was observed, confirming that the cultures were viable and were likely consuming components of the yeast extract only.

### 3.5. General Genomic Features

**Figure 1:**
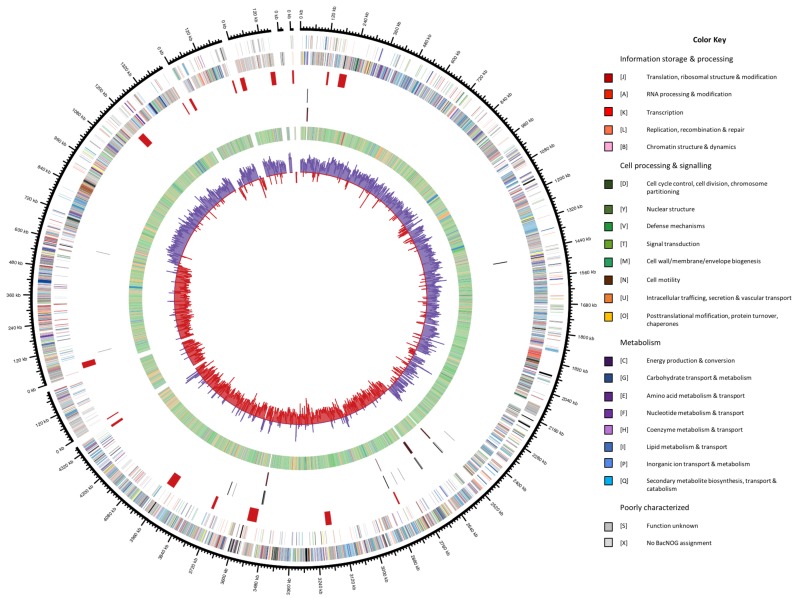
Circular representation of the *V. alkaniphila* L81 genome displaying relevant genome features. Contig order clockwise from top: 0, 5, 2, 6, 3, 7, 1. Circles representing the following (from center to outside): 1, G  +  C skew [(G − C)/(G  +  C) using a 2-kbp sliding window] (blue, positive; red, negative); 2, Taxonomy by Uniref90 top hits; *Firmicutes* (green), *Archaea* (red), *Eukaryota* (yellow), other Bacteria (blue), unknown (orange), no hit to Uniref90 (grey). 3, tRNAs (black); 4, rRNA operons (dark red); 5, Prophages; 6, Coding DNA sequence (CDS) on the reverse strand; 7, CDS on the forward strand. Color coding of CDS was based on COG categories. The figure was build using Circos version. 0.67-1.

The permanent draft assembly of *V. guaymasensis* L81 resulted in 7 contigs with a total of 6.4 Mbp with an average coverage of 75. This places the *V. guaymasensis* L81 genome among the larger sequenced genomes [39,40]. The genome of *V. guaymasensis* L81 represents the first published genome from the genus *Vallitalea*. The GC content of 31.2% is uncharacteristically low for such a large genome [40], with the majority of coding sequences on the leading strand, a common feature in low GC *Firmicutes* [41]. The genome has 6 identical copies of the 16S rRNA gene. There are 17 regions of phage related genes on the genome, and repeated regions in these prophages cause difficulties in genome assembly. Figure 1 shows a circular representation of the genome, illustrating how the contigs are mainly split in phage regions. There are no clear indications of horizontal gene transfer on the genome, however, the low GC content makes transferred regions, tending towards lower GC content than the host genome [42], hard to identify. Table 1 summarizes the major properties of the genome. 

### 3.6. Carbohydrate Metabolism and Transport 

A high percentage of the annotated genes (23.8% of genes in RAST) can be related to the metabolism of carbohydrates, consistent with growth experiments. Moreover, 85 ABC transporters putatively related to carbohydrate transport were identified (Supplementary Table S1). *V. guaymasensis* L81 was shown to grow on arabinose, fructose, glucose, glycerol, galactose, galacturonate, lactate, pectin, polygalacturonate, and ribose in the original characterization of the strain [4]. Additionally, cellobiose, dextrin, lactose, maltose, mannose, palatinose, starch, sucrose, xylan, and xylose were confirmed to support growth in pure culture in this study.

A complete glycolysis pathway was identified, as were all enzymes involved in gluconeogenesis (Supplementary Figure S1 and Table S2). The Entner–Doudoroff pathway and the oxidative part of the pentose phosphate pathway were incomplete, as observed in other Gram-positive bacteria [43]. In addition to anabolic functions, the partial pentose phosphate pathway may serve as a pathway for isomerization and rearrangement of sugars to fuel the glycolysis in *V. guaymasensis* L81. Starch, isomaltulose, cellobiose, dextrin, galactose, lactose, maltose, mannose, palatinose, and sucrose could all be converted to glucose-6-phosphate for the glycolysis pathway using the enzymes listed in Supplementary Table S2, while glycerol enters the glycolysis pathway as 3-phosphoglycerate (Supplementary Figure S1 and Table S2). 

Xylan, arabinoxylan, xylose arabinose, and ribose could enter the glycolysis through the pentose phosphate pathway. The genome also encoded a putative capacity for the degradation of chitin that would enter the glycolysis as fructose-6-phosphate. A putative complete pathway for cellulose degradation to glucose and fructose-6-phosphate was annotated by RAST [27,28,29] (Supplementary Figure S1 and Table S2). The enzyme annotated as an endoglucanase in the cellulose degradation pathway (peg.3524) appears to be a dubious annotation. No carbohydrate binding modules were identified in the protein by dbCAN [33], and it contained several regions of glycine repeats indicative of a phage origin. This leaves the pathway annotation incomplete, though cultivation experiments suggest that it may be functional.

The pyruvate resulting from glycolysis could fuel the citric acid (TCA) cycle; either as oxaloacetate or acetyl-CoA catalyzed by pyruvate carboxylase and pyruvate ferredoxin oxidoreductase, respectively. The TCA cycle is incomplete due to lacking genes for succinyl-CoA synthetase, and probably function as a biosynthesis pathway for amino acids and tetrapyrroles.

Pyruvate can also be directly fermented to lactate by lactate dehydrogenase, or enter the acetyl-CoA pool by pyruvate:ferredoxin oxidoreductase or pyruvate-formate lyase (Supplementary Figure S1 and Table S2), with a concomitant production of formate by the latter enzyme. The acetyl-CoA could be converted to acetate and ethanol in the conventional mixed acid fermentation pathway, to acetate via acetyl phosphate, or to acetate directly (Supplementary Figure S1 and Table S2). Acetyl-CoA can also be fermented to butyrate via two different pathways where the conversion of acetyl-CoA to butyryl-CoA is identical in both pathways, with acetoacetyl-CoA, 3-hydroxybutanoyl-coA and crotonyl-CoA as intermediates (Supplementary Figure S1 and Table S2). Butyryl-CoA can either be coupled with acetate to form butyrate, a reaction catalyzed by butyrate-acetoacetate CoA transferase; or form butyrate via butyryl phosphate, catalyzed by phosphotransbutyrylase and butyrate kinase. Genes for converting butyryl-CoA to butanol were also present in the genome (Supplementary Table S2). Genes encoding pathways leading to propionate, acetone or 2-propanol were not detected.

### 3.7. Protein Metabolism and Transport

*Vallitalea guaymasensis* L81 can grow on peptone and yeast extract as carbon and energy source [4], congruently, the genome encodes proteases and peptidases as well as membrane transport systems for oligopeptides, dipeptides, branched chain amino acids, and polar amino acids (Supplementary Table S2). The genome analysis shows that *V. guaymasensis* L81 is unable to synthesize l-phenylalanine, l-tyrosine, l-histidine, l-arginine, l-isoleucine, l-leucine, l-methionine, l-lysine or l-threonine, and thus rely on acquiring these amino acids from the external environment. Contrarily, genes for the synthesis of l-alanine, l-aspartate, l-glutamate, l-tryptophan, l-glycine, l-aspargine, l-glutamine, l-valine, l-serine, l-cysteine, and l-proline were identified. 

Stickland reactions are used by amino acid degrading *Clostridia*, like *Clostridium sticklandii*, and require amino acid pairs, where one amino acid is reduced and the other oxidized [44]. In methanogenic consortia, methanogens can take the role of the reductive part of the Stickland reaction, removing the need for amino acid pairs [45]. A syntrophic relationship is also favorable in terms of thermodynamics. Low hydrogen pressure is favorable for the amino acid degradation reactions, and for some reactions, such as the degradation of alanine to acetate, syntrophy is a requirement [45,46]. The genomic data of *V. guaymasensis* L81 suggests that amino acids are fermented with protons as electron acceptor, and not degraded via Stickland reactions, as no candidate for an amino acid reductase was identified. The observation was supported by our laboratory experiments where low amounts of hydrogen were produced by pure cultures, and methane by consortia grown on yeast extract and peptone. This is similar to the *Acetoanaerobium pronyense* that was isolated from the Prony hydrothermal vent field [47]. 

The degradation pathways present for l-alanine, l-threonine, l-glycine, and l-serine all lead to pyruvate. The fructoselysine and l-lysine degradation pathway identified, is identical to that previously described for *Intestimonas* strain AF211 [48], where fructoselysine is phosphorylated to fructoselycine-6-phosphate, and subsequently split into l-lysine and glucose-6-phosphate. Glucose-6-phosphate enters the glycolysis, while l-lysine is fermented to acetate and butyrate. 

There are no complete annotated ATP-yielding pathways for histidine and glutamate degradation in this genome, however, for the putative methylaspartate pathway [46,49], only the citramalate lyase was missing. It is possible that an enzyme different from those previously described could catalyze this reaction. 

### 3.8. Alkane Activation and Degradation

*Vallitalea guaymasensis* L81 encodes two putative alkylsuccinate synthases for activation of *n*-alkanes by addition of fumarate [4] (Supplementary Table S2). A complete putative pathway for alkane degradation was however not identified on the genome. The enzymes involved in the carbon skeleton rearrangement and decarboxylation of the methylalkylsuccinic acids resulting from fumarate addition are not conclusively described [50]. An acyl-CoA synthetase has been suggested as a possible candidate for the addition of S-CoA in *D. alkenivorans* AK01 [50]. One putative acyl-CoA synthetase (peg.3802), and two long chain fatty acyl-CoA synthetases (peg.2630, peg.3316) were identified in the *V. guaymasensis* L81 genome. It has been suggested that an enzyme analogous to methylmalonyl-CoA mutase is responsible for the carbon skeleton rearrangement [50,51]. No homolog for a methylmalonyl-CoA mutase was identified on the *V. guaymasensis* L81 genome. Two acetyl-CoA carboxyl transferases (peg.444, peg.4125) that could be involved in decarboxylation of the fatty acyl-CoA prior to β-oxidation were also identified. 

A putative complete pathway for β-oxidation of the fatty acids was identified (Supplementary Figure S1 and Table S2), where three putative acyl-CoA dehydrogenases were identified, of which two were located directly upstream of electron transfer flavoprotein (Etf) complexes (peg.3682, 1149). Moreover, three putative enoyl-CoA hydratases, two 3-hydroxyacyl-CoA dehydrogenases and two 3-ketoacyl-CoA thiolases were identified. 

Regeneration of fumarate is required for this mode of alkane activation and has been shown to occur via the methylmalonyl-CoA pathway in *D. alkenivorans* AK-01 [50]. Of the required enzymes for this pathway, we could not identify homologs for succinyl-CoA synthetase or succinate dehydrogenase in *V. guaymasensis* L81.

### 3.9. Redox-Components

**Figure 2:**
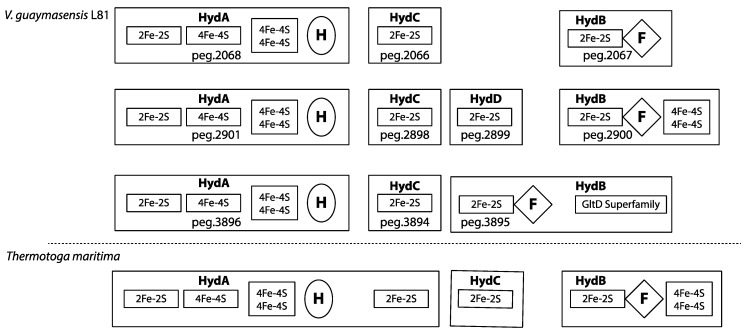
Domain organization of multimeric Fe-Fe hydrogenases. Large boxes represent hydrogenase subunits. The trimeric bifurcating/confurcating hydrogenase from *T. maritima* is shown for comparison. H, H-cluster; 2Fe-2S, cluster binding site; 4Fe-4S, cluster binding site; F, FMN and NAD^+^ binding site. *T. maritima* data from Soboh and co-workers (2004) [61].

Three putative bifurcating/confurcating, multimeric [FeFe] hydrogenases (two trimeric and one tetrameric) were identified in *V. guaymasensis* L81 (Figure 2). The three multimeric [FeFe] hydrogenases have conserved regions similar to the trimeric bifurcating/confurcating hydrogenase from *Thermotoga maritima* [52]. The α-subunits (HydA) of all three enzymes contained the H-cluster binding domain and the conserved upstream FeS clusters similar to the *T. maritima* α-subunits [53,54]. However, all three α-subunits of the *V. guaymasensis* L81 multimeric hydrogenases are lacking the C-terminal [2Fe-2S] binding domain found in *T. maritima.* Since the fourth subunit (HydD) (peg.2899) of the tetrameric [FeFe] hydrogenases of *V. guaymasensis* strain L81carries a [2Fe-2S]-cluster binding domain similar to the C-terminal [2Fe-2S]-cluster domain in the *T. maritima* α-subunit, this isoenzyme appears most similar in architecture to the *T. maritima* trimeric enzyme. Moreover, a γ-subunit (HydC) with a 2Fe-2S domain was part of each hydrogenase. The β-subunits (HydB) in *V. guaymasensis* L81 carry the FMN/NADH-binding domain and the [2Fe-2S]-cluster binding domain as identified in the *T. maritima* enzyme. However, the two-[4Fe-4S] binding domain is only conserved in the tetrameric enzyme (peg.2898, 2899, 2900, 2901). This domain is absent in the first trimeric hydrogenase (peg.2066, 2067, 2068), and substituted by an enlarged β-subunit (peg.3895) with a GltD binding domain after the FMN/NADH-binding domain in the second trimeric hydrogenase (peg.3894, 3895, 3896), suggesting a second NAD(P)H binding site. 

One monomeric [FeFe] hydrogenase (peg.4378) was classified by HydDB [36] as belonging to group C1, linked to a sensor histidine kinase (peg.4376). Group C1 hydrogenases are postulated to be involved in hydrogen sensing, and regulation of transcription of other hydrogenases [55,56].

Two [FeFe] hydrogenases (peg.96 and 98) were classified by HydDB as belonging to group C3 and group B hydrogenases respectively. The function of group C3 hydrogenases remain undescribed, but the members of this group are co-transcribed with hydrogen evolving hydrogenases associated with fermentation, and appear to regulate the transcription of these hydrogenases [55,56,57,58,59]. The function of group B hydrogenases remains unconfirmed, but indirect evidence suggests they are involved in hydrogenogenic fermentation, coupling the reoxidation of reduced ferredoxin to hydrogen evolution [55,57,60].

Elements of a putative [NiFe] hydrogenase were identified, consisting of HypE (peg.4591), HypD (peg.4592), HypC (peg.4593), HypF (peg.4594), HyaD (peg.4596), a [NiFe] hydrogenase large subunit (peg.4597), and a [NiFe] hydrogenase small subunit (peg.4599). The large and small subunits were annotated by RAST and HydDB as belonging to the [NiFe] group 1 uptake hydrogenases.

The two electron-transfer flavoprotein (Etf) complexes encoded in the genome, are directly linked to the butyryl-CoA/acyl-CoA dehydrogenase complexes. One of the Etf β-subunits (peg.3683) contains the NADH and FAD binding site marker sequences for a bifurcating Etf [62,63]. 

The genome encodes both a V-type ATPase and a F-type ATPase. F-ATPases generate ATP via proton translocation, while V-ATPases utilize ATP to translocate protons across membranes [64]. Contrary to F-ATPases, the function of V-ATPases is not reversible, which renders them unable to generate ATP. The function of V-ATPases is thus solely to produce a proton motive force [64], and the presence of a V-ATPase makes energy generation by electron transport phosphorylation probable in *V. guaymasensis* L81. 

**Figure 3:**
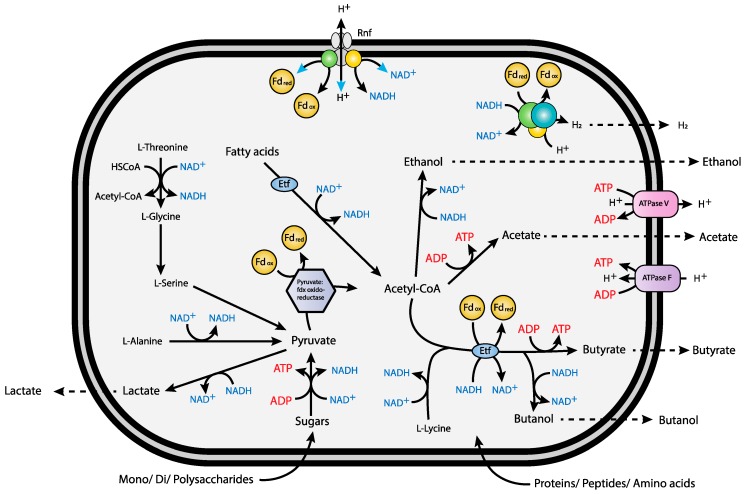
Overview of the main metabolic pathways and energy production in *V. guaymasensis* L81. The overall scheme for energy conservation in *V. guaymasensis* L81 is simple. Proton gradient generation is probably done by Rnf mediated Fd_red_ oxidation. In the absence of a terminal electron acceptor or corresponding electron transport chains, hydrogen generated by cytoplasmic hydrogenases is probably produced as a non-respiratory, fermentative mechanism. As Fd_red_ is needed for H_2_ generation at higher partial pressure, the bifurcating/confurcating [FeFe] hydrogenases may be capable of conserving energy, coupling oxidation of Fd_red_ and NADH [65].

The genome also encodes a proton/sodium ion-translocating Rnf (*Rhodobacter* nitrogen fixation) complex (peg.4370–4375), catalyzing the reversible oxidation of reduced ferredoxin with NAD^+^ [9] (Figure 3).

The genome of *V. guaymasensis* L81 lacks homologues corresponding to the complexes of the mitochondrial respiratory chain, including genes encoding a NADH:ubiquinone oxidoreductase (Nuo) complex. In addition, genes corresponding to respiratory cytochromes are absent. 

### 3.10. Polyketid Synthesis

The antiSMASH analysis revealed 9 different gene clusters potentially involved in the synthesis of polyketids (Supplementary Table S2). Four of the clusters showed similarities with previously described biosynthetic clusters, as listed below, while five clusters contained genes for non-ribosomal peptide synthases and polyketide synthases without showing similarities to previously described polyketide synthesis clusters.

Cluster 3 showed similarity with several previously described biosynthetic clusters: A polyketide synthase (peg.4909) and a 3-hydroxybutyryl-CoA dehydrogenase (peg.4915) showed similarity to genes from zwittermycin A [66], paenilamicin [67], and colibactin [68] biosynthetic clusters. A similar 3-hydroxybutyryl-CoA dehydrogenase is also found in the pellasoren biosynthetic cluster [69], along with a butyryl-CoA dehydrogenase similar to peg.4913 from the *V. guaymasensis* L81 cluster 3. The cluster also contains genes encoding a lanthibiotic transport permease protein (peg.4929), and a lanthibiotic transport ATP-binding protein (peg.4931) similar to those found in the ericin A [70], entianin [71], and subtilin [72] biosynthetic clusters. The lacticin 481 [73,74] biosynthetic cluster contains genes encoding proteins similar to the lanthibiotic transport ATP-binding protein (peg.4931), and a transposase (peg.4926) from cluster 3. The low similarity, in terms of homologous proteins per cluster, with previously described biosynthetic clusters makes it difficult to predict the nature of the polyketide produced by cluster 3.

Cluster 5 showed similarity with several previously described biosynthetic clusters, namely those for nosperin [75], thiomarinol [76] kalimantacin [77], thailandamide [78,79,80], bacillaene [81,82,83], elansolid [84], bongkrekic acid [85], calyculin [86], cylindrocyclophane [87], and carbamidocyclophane [88]. Apart from two putative polyketide synthases (peg.1164 and 1168), the similarity lies in a string of 8 small proteins that are represented in various configurations in the mentioned biosynthetic clusters. The hydroxymethylglutaryl-CoA synthase (peg.3083 and 3085) has homologs in all the clusters, while the 3-oxoacyl synthase (peg.3084) has homologs in all clusters apart from the one for calyculin. A methylglutaconyl-CoA hydratase (peg.3082) was homologous with enoyl-CoA hydratases from the thiomarinol, kalimantacin, thailandamide, bacillaene and calyculin clusters, while the enoyl-CoA hydratase (peg.3081) was homologous with a second enoyl-CoA in the kalimantacin, thailandamide, bacillaene, elansolid and calyculin clusters. The enoyl-CoA from cluster 5 was also homologous to enoyl-CoA hydratases in the nosperin and bongkrekic acid clusters. The acyl carrier protein (peg.3086) had homologs in the thiomarinol, kalimantacin thailandamide, bacillaene, and elansolid clusters. A malonyl-CoA acyl carrier protein had homologs in the nosperin, kalimantacin, bacillaene, elansolid, and bongkrekic acid clusters. The only homolog for the acetyl-CoA carboxyl transferase (peg.3090) was found in the thailandamide cluster. 

Cluster 6 harbor genes homologous to LanB, LanC and the ABC-transporter, LanT, of the type I lanthibiotic synthesis pathway [89]. The gene cluster was most similar to the penisin biosynthetic gene cluster from *Penibacillus eheimensis* A3 [90]. Homologs for the precursor, Lan A (PenA), and the serine protease, LanP, were not identified, so it is uncertain if lanthibiotics are produced by the strain. LanP is also missing from the *P. eheimensis* A3 penisin cluster, demonstrating that LanP is non-essential for the synthesis of functional lanthibiotics. The *P. eheimensis* A3 penisin cluster contains two additional proteins, PenD and PenR. PenD has been suggested as a dehydratase, and PenR as a possible tanscriptional regulator [90]. The genome of *V. guaymasensis* L81 contains three homologs to PenD (peg.331, 361, 4587) and one homolog to PenR (peg.359), however, neither are encoded in the same cluster as LanB, LanC, and LanT, and thus, uncertain to be involved in polyketide synthesis. Finally, except for two genes similar to the ABC transporter genes eqbK and eqbL found in the equibactin biosynthetic cluster from *Streptococcus equi* [91], cluster 9 showed no similarities with other described biosynthetic clusters.

### 3.11. Intracellular Compartments

SEM micrographs revealed the presence of internal membrane structures in the cells, however, the function of these structures remains unknown [4]. Interestingly, the genome contains genes for the formation of a combined propanediol utilizing (PDU) and ethanolamine utilizing (EUT) type of bacterial microcompartments (BMC) [92,93]. These processes have to be sequestered in compartments since the intermediate, propionaldehyde from propanediol degradation, is mutagenic, and hence toxic to the cells [94,95,96,97]. Furthermore, acetaldehyde from ethanolamine degradation is volatile and needs to be sequestered to not escape the cell [95,96,98]. Present evidence suggests that these PDU/EUT fusion loci produce separate PDU and EUT BMCs [92]. PDU microcompartments have also been shown to be involved in bacterial degradation of rhamnose and fucose [99,100]. The function of BMCs in *V. guaymasensis* L81 is uncertain, as no propanediol oxidoreductase was identified. However, an alpha-l-fucosidase is encoded in the BMC locus in *V. guaymasensis* strain L81, indicating a role in fucose degradation. This would require a different enzyme to perform the part of the missing propanediol oxidoreductase. 

## 4. Discussion

Cultivation experiments and genome analysis have revealed that *V. guaymasensis* L81 has the capacity to fulfill multiple metabolic roles in its environment at Loki’s castle hydrothermal vent field. Fermentation of mono-, di- and polysaccharides occurs via the glycolysis pathway and may lead to a formation of acetate and H_2_, which in co-cultures supports the growth of methanogenic partners and subsequent CH_4_-production. *Vallitalea guaymasensis* strain L81 also encodes a capacity for the formation of lactate, ethanol, butyrate, or butanol as fermentation products. Amino acids are predicted to be oxidatively converted to acetate, CO_2_ (and NH_4_^+^) with concomitant interspecies hydrogen transfer to methanogenic partners [101]. 

During the fermentation of saccharides and amino acids like l-Histidine, l-threonine and l-alanine, Fd_red_ is produced by oxidation of pyruvate to acetyl-CoA and CO_2_ in a reaction catalyzed by pyruvate:ferredoxin oxidoreductase, while reduced NADH is formed in multiple redox-reactions (Figure 2, Supplementary Figure S1). The formation of H_2_ as an intermediate in these fermentation reactions is predicted to be facilitated by cytoplasmic bifurcating/confurcating hydrogenases. The presence of the GltD-domain on one of the hydrogenase subunits (HydB) may indicate a specific link to the oxidation or assimilation of glutamate. In butyrogenic fermentation, the endergonic reduction of ferredoxin with NADH could be coupled to the exergonic reduction of crotonyl-CoA to butyryl-CoA catalyzed by a putative butyryl-CoA/Etf complex. The two Etf-complexes encoded in the genome, are directly linked to the butyryl-CoA/acyl-CoA dehydrogenase complexes. Moreover, one of the Etf β-subunits contain the marker sequences for a bifurcating Etf [62,63]. This suggests that the *V. guaymasensis* strain L81 can use electron bifurcation via the electron transferring flavoprotein-butyryl-CoA dehydrogenase (Etf-Bcd) complex to generate reduced ferredoxin during acetyl-CoA fermentation to butyrate [62,102,103,104,105]. The non-bifurcating Etf complex is directly linked to an acyl-CoA dehydrogenase, and in close proximity to a long-chain fatty acid CoA ligase and a 3-hydroxyacyl-CoA dehydrogenase (Supplementary Table S1). This indicates that it may be involved in the conversion of acyl-CoA to enoyl-CoA in the β-oxidation cycle, as described for *Smithella* spp. [15]. The crotonyl-CoA formed in the fermentation of fructoselysine or lysine may be disproportionated to acetate, butyrate, and H_2_, where Fd_red_ for the H_2_ formation is, as for the butyrogenic fermentation, generated by electron bifurcation with crotonyl-CoA and NADH. Since *V. guaymasensis* L81 encodes a Rnf complex and a V-type ATPase, re-oxidation of Fd_red_ may also be catalyzed by the encoded Rnf-complex, representing an alternative route for energy conservation. The energy difference between reduced ferredoxin and NAD^+^ of about 200 mV is proposed to be used by the Rnf-complex to generate an electrochemical H^+^ or Na^+^ gradient [103,106]. In *C. ljungdahlii* the Rnf complex plays a crucial role in pumping protons out of the cell membrane for energy conservation during acetogenic, autotrophic growth, but it was also suggested to contribute to ATP synthesis during heterotrophic growth on fructose by generating a proton gradient [107]. The Rnf complex may also contribute to the appropriate NADH/Fd_red_ ratio that will be affected by biosynthesis and the oxidation state of the growth substrates. The direct association of a putative sensory hydrogenase (peg.4378) and a sensory histidine kinase (peg.4376) to the Rnf complex (peg.4370–4375) indicates that the activity of the Rnf complex may be regulated by extracellular H_2_ concentrations.

As previously reported, *V. guaymasensis* L81 appears to degrade a wide spectrum of alkanes as assessed by whole-oil gas chromatography [4]. In contrast to a confirmed capacity to perform methanogenic syntrophic growth on protein-rich compounds and saccharides, a complete pathway for utilization of alkanes was not identified in the genome analysis. Growth on alkanes requires enzymes for the activation of alkanes and for β-oxidation of fatty acid intermediates [51]. In the absence of external electron acceptors, such as iron, nitrate or sulfate, this process is an obligate syntrophic reaction, and hydrogenases for H_2_ production are required [108,109,110]. Interestingly, *V. guaymasensis* L81 appears to have the capacity for reverse electron transport driven H_2_-production that would be required for syntrophic fermentation of hydrocarbons to methane. ATP may be formed via substrate level phosphorylation in the conversion of acetyl-CoA intermediates to acetate during the oxidation of fatty acids. This ATP could then be used for creating a proton gradient by pumping protons to the periplasmic space. The resulting proton-gradient could again support Rnf-driven ferredoxin reduction coupled to reoxidation of NADH formed in the oxidation of 3-hydroxybutanoyl to acetoacetyl-CoA. This Fd_red_ could subsequently support H_2_-production by one or more of the putative multimeric bifuricating [FeFe] hydrogenases in *V. guaymasensis* L81. Moreover, this Fd_red_ could feed the bifuricating Bcd-Etf complex, coupling the endergonic oxidation of butyryl-CoA to crotonyl_CoA with the exergonic reduction of NAD^+^ with Fd_red_. In the fermentation pathway of *n*-alkanes an enzyme homologous to methylmalonyl-CoA mutase has been proposed to be responsible for carbon skeleton rearrangement of the methylalkylsuccinates resulting from fumarate activation [50,51]. As no enzyme homologous to methylmalonyl-CoA mutase was identified in the *V. guaymasensis* L81 genome, we cannot say if this reaction is plausible in this organism. Altogether, there is potentially a metabolic capacity for syntrophic methanogenic growth on alkanes in *V. guaymasensis* L81. However, significant methane-production was not observed during growth with a selection of *n*-alkanes as substrate, and further work is needed to confirm this metabolism. Another possibility is that the degradation of crude oil-components observed in *V. guaymasensis* L81 [4] is a result of an unspecific activation, and not a full hydrocarbon metabolism directly yielding energy for the cell. It has previously been demonstrated that the hydrocarbon activation enzymes show a relaxed specificity, particularly those for *n*-alkanes, and activate a broader range of hydrocarbons than can be fully metabolized by the cell [111,112]. One possible function of this broad-spectrum activation is detoxification [111]. Hydrocarbons are toxic to microorganisms and can diffuse over the cell membrane [111]. Jarling and co-workers (2015) proposed that the transformation of hydrocarbons to di-acids by activating enzymes, reduces the toxic effect, as the two negative charges of the di-acid prevents the molecule from penetrating and disrupting the cell membrane [111]. This potentially allows the hydrocarbon degrading bacteria to grow closer to an oil-water interface than they otherwise could. The ability to detoxify is also beneficial to microorganisms not utilizing hydrocarbons for energy, as it enables them to grow in hydrocarbon-contaminated environments [111]. Hydrocarbons like methane, acetylene, ethylene, ethane, and butane have been detected in venting fluids from Loki’s castle [7], demonstrating that this may be a useful function in the system. As the venting fluids at Loki’s castle also contain hydrogen concentrations of up to 5.5 mmol kg^−1^ [7], this would shift obligately syntrophic, low energy reactions, such as hydrocarbon degradation and fermentation of amino acids such as alanine, towards being unfavorable. These reactions are thus more probable to occur in surrounding sediments.

**Figure 4:**
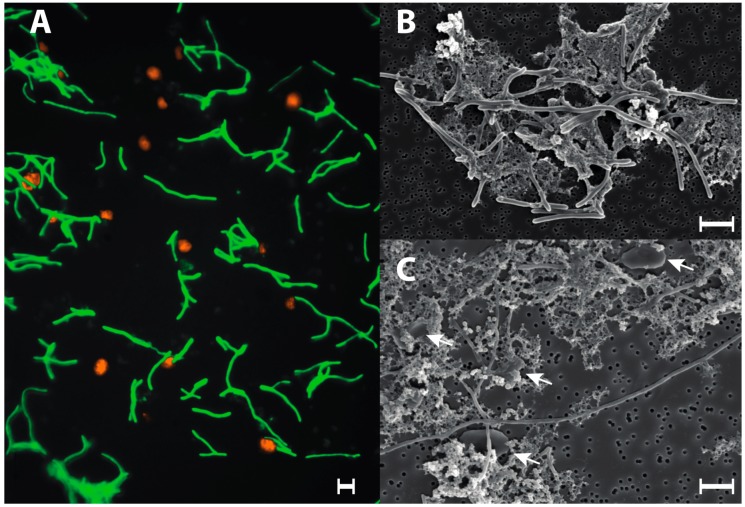
(**A**) Fluorescent in situ hybridization image of a stationary phase co-culture of *V. guaymasensis* L81 (labeled in green) and *M. limicola* (labeled in orange), utilizing glucose as substrate for growth; (**B**) Scanning electron micrograph of a stationary phase co-culture of *V. guaymasensis* L81, and *M. limicola*, given glucose; (**C**) Scanning electron micrograph (SEM) of a stationary phase co-culture of *V. guaymasensis* L81, and *M. limicola*, given crude oil. Arrows mark cells of *M. limicola*. Scale bars: 2 µm.

Successful syntrophic interactions rely on effective transfer of metabolites, such as hydrogen and formate, between partner organisms [113,114,115,116,117,118]. The flagellum proteins FliC and FliD have been shown to be important in the adherence of *Pelotomaculum thermopropionicum* to its methanogenic partners *Methanothermobacter thermautotrophicus* and *Methanosaeta thermophila* during syntrophic growth [119,120], and FliD was also shown to enhance the methanogenic activity of *M. thermautotrophicus* [120]. A complete set of genes for flagellar assembly was observed in *V. guaymasensis* L81 indicating that a similar mechanism could occur in this organism. When studied using light microscopy, cells of *V. guyamasensis* L81 and *M. limicola* were observed both free-living and clumped together in aggregates of cells and precipitated iron sulfides. A potential for biofilm formation was observed using SEM and FISH analyses in the stationary phase of co-cultures grown on glucose, but no flagella or pili were observed (Figure 4). The SEM investigation indicates the formation of an extracellular substance that likely aids cell aggregation (Figure 4). Cells generally showed a higher tendency to aggregate during growth on unfavorable substrates, and with low substrate concentrations. There are several other features revealed through the genome analysis that point to interactions with other organisms in the ecosystem. The genome of *V. guaymasensis* L81 encodes genes for the synthesis of various polyketides that could be utilized as means of keeping competitors at bay. Genes for vancomycin resistance and aminoglycoside resistance were also identified. These may be useful as protection from antibiotics produced by the cell itself, or as defense against competitors. The prophages present on the genome reflect the constant impact of viruses on the microbial community.

The varied metabolic toolbox, coupled with polyketid biosynthesis clusters, and multiple prophages, makes *V. guaymasensis* L81 an interesting subject for further studies.

## 5. Conclusions

*V. Guaymasensis* L81 is a versatile organism, with the ability to utilize a wide range of carbohydrates and peptides, both in pure culture, and in co-cultures with a methanogenic partner. The observed hydrocarbon degradation facilitated by the strain is proposed to be unspecific activation, possibly as a detoxification mechanism, rather than energy metabolism. The genome infers an ability to degrade complex polymers such as chitin and xylan. Along with 9 putative polyketide synthesis clusters, this makes the strain interesting from an industrial perspective.

## Figures and Tables

**Table 1:** General genome features.

Category	RAST	IMG-ER
Genome size	6419149 bp	6419149 bp
Contigs	7	7
GC content	31.2%	31.2%
Coding sequences	5628	5651
RNA genes	67 (1.2%)	121 (2.1%)
rRNA genes	11	17
5S rRNA		6
16S rRNA		6
23S rRNA		5
tRNA genes	56	56
Other RNA genes		48
Genes with function prediction	3820 (67.87%)	4043 (70.05%)
Genes without function prediction	1808 (32.13%)	1608 (27.86%)

## Supplementary Materials

The following are available online at http://www.mdpi.com/2076-2607/6/3/63/s1, Figure S1: Core metabolic reactions, Table S1: Phenotypic properties important for reclassification, Table S2: Annotations related to Figure S1.
