# Supplementary material for: Genome Analysis of Vallitalea guaymasensis Strain L81 Isolated from a Deep-Sea Hydrothermal Vent System
Source: Microorganisms. 2018 Jul 4;6(3):63. doi: 10.3390/microorganisms6030063 (PMC6163223; doi:10.3390/microorganisms6030063)
Supplement: Supplementary file 1 [file microorganisms-06-00063-s001.zip › Supplementary table 1.pdf]

Supplementary Table 1. Differential phenotypic characteristics between *A. alkaniphila* strain L81^{T} and strain PC1 and the closest relatives within *Vallitalea spp.* and *Natranaerovirga spp*.

Strains: 1. *Abyssivirga alkaniphila* L81^{T} (this study); 2. *Abyssivirga alkaniphila* PC1 (this study); 3. *Vallitalea guaymasensis* Ra1766G1^{T} (this study); 4. *Vallitalea pronyensis* FatNI3^{T} (Ben Aissa et al. 2014); 5. *Natranaerovirga pectinivora* AP3^{T} (Sorokin et al. 2012); 6. *Natranaerovirga hydrolytica* APP2^{T} (Sorokin et al. 2012). Positive result:+, negative result: -, Weak positive result: (+), No Data: ND

**Characteristic**	**1**	**2**	**3**	**4**	**5**	**6**
Gram stain	+	+	-	+	+	+
String test	+	+	+	ND	ND	ND
Optimum Temperature (°C)	37[¤]	37	30-35[#]	30	43	45
Spores	-	-	-	+	+	+
Motility	+	+	+	+	+[¤]	+[¤]
Flagella	ND[§]	Peritrichous	Polar monotrichous	ND	ND	ND
Utilization of:						
Acetate	-	-	-	-	+	+
Arabinose	+	+	+	-	ND	ND
Fructose	+	-	(+)	+	ND	+
Pectin	+	-	+	ND	+	+[¤]
Pyruvate	-	-	+	ND	ND	ND
Rhamnose	(+)	(+)	-	+	ND	ND

[§]Flagella were not detected by the staining method; however, motile cells have been observed using light microscopy.

[#]Data from (Lakhal et al. 2013).

[¤]Data from (Schouw et al. 2015).
