# Supplementary material for: Genome Analysis of Vallitalea guaymasensis Strain L81 Isolated from a Deep-Sea Hydrothermal Vent System
Source: Microorganisms. 2018 Jul 4;6(3):63. doi: 10.3390/microorganisms6030063 (PMC6163223; doi:10.3390/microorganisms6030063)
Supplement: Supplementary file 1 [file microorganisms-06-00063-s001.zip › Supplementary Table 2.pdf]

The table contains proteins mentioned in the article, or represented in supplementary figure 1. Reaction numbers correspond to numbers in supplementary figure 1. Annotations and gene numbers are from RAST.

Reaction nr.	Pathway	Gene	Gene number in RAST
1	Glycolysis and Gluconeogenesis	Glucokinase (EC 2.7.1.2)	1408, 1611, 2184, 2813
2	Glycolysis and Gluconeogenesis	Glucose-6-phosphate isomerase (EC 5.3.1.9)	2206, 2230
3	Glycolysis and Gluconeogenesis	6-phosphofructokinase (EC 2.7.1.11)	1595, 2201, 2347
4	Glycolysis and Gluconeogenesis	Fructose-1,6-bisphosphatase, Bacillus type (EC 3.1.3.11)	18, 3733, 3734, 3735, 3737
5	Glycolysis and Gluconeogenesis	Fructose-bisphosphate aldolase class II (EC 4.1.2.13)	449, 489, 943, 999, 1076, 2204, 3206, 4429
6	Glycolysis and Gluconeogenesis	Triosephosphate isomerase (EC 5.3.1.1)	2202, 5040
7	Glycolysis and Gluconeogenesis	NAD-dependent glyceraldehyde-3-phosphate dehydrogenase (EC 1.2.1.12)	5042
8	Glycolysis and Gluconeogenesis	Phosphoglycerate kinase (EC 2.7.2.3)	5041
9	Glycolysis and Gluconeogenesis	Non-phosphorylating glyceraldehyde-3-phosphate dehydrogenase (NADP) (EC 1.2.1.9)	49
10	Glycolysis and Gluconeogenesis	2,3-bisphosphoglycerate-independent phosphoglycerate mutase (EC 5.4.2.1)	5039
11	Glycolysis and Gluconeogenesis	Enolase (EC 4.2.1.11)	5038
12	Glycolysis and Gluconeogenesis	Pyruvate kinase (EC 2.7.1.40)	540
13	Glycolysis and Gluconeogenesis	Phosphoenolpyruvate synthase (EC 2.7.9.2)	2517, 2533
14	Glycolysis and Gluconeogenesis	Pyruvate,phosphate dikinase (EC 2.7.9.1)	3875
15	Pentose phosphate pathway	Ribulose-phosphate 3-epimerase (EC 5.1.3.1)	3983
16	Pentose phosphate pathway	Transketolase (EC 2.2.1.1)	2937
16	Pentose phosphate pathway	Transketolase, N-terminal section (EC 2.2.1.1)	376
16	Pentose phosphate pathway	Transketolase, C-terminal section (EC 2.2.1.1)	377
19	Pentose phosphate pathway	Ribose 5-phosphate isomerase B (EC 5.3.1.6)	2295
20	Pentose phosphate pathway	Ribose-phosphate pyrophosphokinase (EC 2.7.6.1)	2360, 3104
21	Pentose phosphate pathway	Transaldolase (EC 2.2.1.2)	13, 78
22	TCA cycle	Citrate synthase (si) (EC 2.3.3.1)	335, 3962
23	TCA cycle	Aconitate hydratase (EC 4.2.1.3)	334, 3937
24	TCA cycle	Isocitrate dehydrogenase (EC 1.1.1.41)	332, 3938
25	TCA cycle	2-Oxoglutarate ferredoxin oxidoreductase (EC 1.2.7.3)	3297, 3298, 3299, 3300
26	TCA cycle	Fumarate hydratase (EC 4.2.1.2)	2140, 2141
27	TCA cycle	Malate dehydrogenase (EC 1.1.1.37)	5265
28	TCA cycle	Oxaloacetate decarboxylase, alpha subunit (EC 4.1.1.3)	4129
28	TCA cycle	Oxaloacetate decarboxylase, beta subunit (EC 4.1.1.3)	4128
30	TCA cycle	NADP-dependent Malic enzyme (EC 1.1.1.40)	5265
31	TCA cycle	Phosphoenolopyruvate carboxykinase (EC 4.1.1.49)	2172
32	Pyruvate metabolism	Pyruvate:ferredoxin oxidoreductase, delta subunit (EC 1.2.7.1)	1915
32	Pyruvate metabolism	Pyruvate:ferredoxin oxidoreductase, alpha subunit (EC 1.2.7.1)	1914
32	Pyruvate metabolism	Pyruvate:ferredoxin oxidoreductase, beta subunit (EC 1.2.7.1)	1913, 2003
32	Pyruvate metabolism	Pyruvate:ferredoxin oxidoreductase, gamma subunit (EC 1.2.7.1)	1916, 2004
36	Pyruvate metabolism	D-Lactate dehydrogenase (EC 1.1.1.28)	539
37	Pyruvate metabolism	Pyruvate formate lyase (EC 2.3.1.54)	600, 1934, 3846, 4626
38	Acetyl-coA metabolism and fermentation	Phosphate acetyltransferase (EC 2.3.1.8)	3996
39	Acetyl-coA metabolism and fermentation	Acetate kinase (EC 2.7.2.1)	3997
40	Acetyl-coA metabolism and fermentation	Acetyl-coA synthetase (ADP forming) (EC 6.2.1.13)	3802
41	Acetyl-coA metabolism and fermentation	Aldehyde dehydrogenase (EC 1.2.1.3)	1435
42	Acetyl-coA metabolism and fermentation	Alcohol dehydrogenase (EC 1.1.1.1)	450, 493, 551, 1162, 1986, 2021, 2114, 4937
43	Acetyl-coA metabolism and fermentation	Acetaldehyde dehydrogenase (EC 1.2.1.10)	450, 493, 2021, 2114
44	Acetyl-coA metabolism and fermentation	Acetyl-coA acetyltransferase (EC 2.3.1.9)	996, 4817
45	Acetyl-coA metabolism and fermentation	3-hydroxybutyryl-coA dehydrogenase (EC 1.1.1.35, EC 1.1.1.157)	1143, 4915
46	Acetyl-coA metabolism and fermentation	3-hydroxybutyryl-coA dehydratase (EC 4.2.1.55)	3681

47	Acetyl-coA metabolism and fermentation	Butyryl-coA dehydrogenase (EC 1.3.8.1)	1149, 3682, 4913
49	Acetyl-coA metabolism and fermentation	Butyrate kinase (EC 2.7.2.7)	4112
50	Acetyl-coA metabolism and fermentation	Butyrate-acetoacetate-coA transferase, subunit alpha (EC 2.8.3.9)	998, 5205
50	Acetyl-coA metabolism and fermentation	Butyrate-acetoacetate-coA transferase, subunit beta (EC 2.8.3.9)	997, 5206
51	Acetyl-coA metabolism and fermentation	Phosphotransbutyrylase (EC 2.3.1.9)	4113
52	Fructose to glycolysis	Fructokinase (EC 2.7.1.4)	654, 1622
1	Glucose to glucose-6P	Glucokinase (EC 2.7.1.2)	1408, 1611, 2184, 2813
54	Lactose to glucose + galactose	Beta-galactosidase (EC 3.2.1.23)	358, 712, 1354, 1362, 1512, 2246, 2248, 2477, 2808, 3799, 3810, 3811, 4114, 4417, 4427, 4637, 5112
55	Galactose to galactose-1P	Galactokinase (EC 2.7.1.6)	1903
56	Galactose-1P to glucose-1P	Galactose-1-phosphate uridylyltransferase (EC 2.7.7.10)	1901
57	Glucose-1P to Glucose-6P	Phosphoglucomutase (EC 5.4.2.6)	865, 2089
68	sucrose to fructose + glucose, maltose to 2xglucose	Alpha-glucosidase (EC 3.2.1.20)	721
59	sucrose to fructose + glucose-1P	Sucrose phosphorylase (EC 2.4.1.7)	1620
60	maltose to glucose + glucose-1P	Maltose phosphorylase (EC 2.4.1.8)	1510, 2093, 2793, 2794, 3456, 3834
		4-alpha-glucanotransferase (amylomaltase) (EC 2.4.1.25)	1746
61	palatinose (isomaltulose) to glucose	oligo-1,6-glucosidase (EC 3.2.1.10)	1608
62	mannose-6P to fructose-6P	mannose-6-phosphate isomerase (EC 5.3.1.8)	1614, 1792, 2197
63	Cellobiose	Beta-glucosidase (EC 3.2.1.21)	986, 3151, 5078, 5190
64	Cellulose	Endoglucanase (EC 3.2.1.4)	3524
65	starch, glucogen	Glycogen phosphorylase (EC 2.4.1.1)	1726
66	starch, glucogen	1,4-alpha-glucan branch enzyme (EC 2.4.1.18)	323
67	Starch	Alpha-amylase (EC 3.2.1.1)	4944
68	starch	Alpha-glucosidase (EC 3.2.1.20)	721
69	Dextrin	Glucoamylase (EC 3.2.1.3)	1591
70	xylan	Alpha-xylosidase (EC 3.2.1.-)	1739, 4625
71	xylan	Alpha-arabinofuranosidase (EC 3.2.1.55)	2500, 4416
72	xylan	Endo-1,4-beta-xylanase (EC 3.2.1.8)	513, 2483
73	Xylose	xylose isomerase (EC 5.3.1.5)	4685
74	Xylose	xylulose kinase (EC 2.7.1.17)	940, 3432, 4683, 5091
75	Ribulose	Ribulokinase (EC 2.7.1.16)	1360, 2663
76	L-ribulose	L-ribulose-5-phosphate 4-epimerase (EC 5.1.3.4)	2664
77	Ribose	Ribokinase (EC 2.7.1.15)	1431, 2018, 2843
78	Arabinose	Arabinose isomerase (EC 5.3.1.4)	2662
79	Rhamnose	Rhamnulokinase (EC 2.7.1.5)	598, 3245
80	Rhamnose	Fuculose-1-phosphate aldolase (EC 4.1.2.17)	594
42	Lactaldehyde to lactate (from rhamnose)	Aldehyde dehydrogenase (EC 1.2.1.3)	1435
	Fucose	Fucose permease	3221
	Fucose	alpha-L-fucosidase (EC 3.2.1.51)	157, 1328
	Fucose	L-fucose mutarotase	406
	Fucose	L-fucose isomerase (EC 5.3.1.25)	1381
90	Lysine degradation	Fructoselysine-6-kinase	3480
91	Lysine degradation	Fructoselysine-6-P-deglycase	3477, 3486
81	Lysine degradation	Lycine decarboxylase (EC 4.1.1.18)	2157
82	Lysine degradation	Lycine 2,3-aminomutase (EC 5.4.3.2)	5062, 5208
83	Lysine degradation	L-beta-lysine 5,6-aminomutase alpha subunit (EC 5.4.3.3)	5211
83	Lysine degradation	L-beta-lysine 5,6-aminomutase beta subunit (EC 5.4.3.3)	5212
84	Lysine degradation	L-erythro-3,5-diaminohexanoate dehydrogenase (EC 1.4.1.11)	5207
85	Lysine degradation	3-keto-5-aminohexanoate cleavage enzyme (EC 2.3.1.247)	5204
86	Lysine degradation	3-aminobutyryl-CoA ammonia-lyase (EC 4.3.1.14)	5203
92	Histidine degradation	Histidine ammonia-lyase (EC 4.3.1.3)	478
93	Histidine degradation	Urocanate hydratase (EC 4.2.1.49)	477
94	Histidine degradation	Imidazolonepropionase (EC 3.5.2.7)	475
95	Histidine degradation	Glutamate formiminotransferase	476

87	Glutamate degradation	Glutamate dehydrogenase (EC 1.4.1.2)	2721
88	Glutamate degradation	Glutamate dehydrogenase (EC 1.4.1.4)	2459
103	Glutamate degradation	Methylaspartate mutase (EC 5.4.99.1)	5247, 5248, 5249
104	Glutamate degradation	Methylaspartate ammonia -lyase (EC 4.3.1.2)	5250, 5251
105	Glutamate degradation	Fumarate hydratase I (EC 4.2.1.2)	2140, 2141
96	Threonine degradation	Threonine dehydrogenase (EC 1.1.1.103)	1336, 2821, 739
97	Threonine degradation	2-amino-3-ketobutyrate coenzyme A ligase(EC 2.3.2.29)	2820
98	Glycine cleavage system	Glycine dehydrogenase P1 (EC 1.4.4.2)	4692
98	Glycine cleavage system	Glycine dehydrogenase P2 (EC 1.4.4.2)	4691
99	Glycine cleavage system	Aminomethyltransferase (EC 2.1.2.10)	4694
100	Glycine cleavage system	Dihydrolipoamide dehydrogenase (EC 1.8.1.4)	949, 4690
101	Glycine cleavage system	Glycine cleavage system protein H	4693
102	Glycine degradation	Serine hydroxymethyltransferase (EC 2.1.2.1)	4741
89	Serine degradation	Serine dehydratase (EC 4.3.1.17)	2156, 2157
106	L-Alanine netabolism	Alanine dehydrogenase (EC 1.4.1.1.)	4688
107	Glycerol	Aldehyde reductase (EC 1.1.1.2, 1.1.1.21)	978, 945
108	Glycerol	Aldehyde dehydrogenase (EC 1.2.1.3)	1435
109	Glycerol	Glycerate kinase (EC 2.7.1.31)	575, 938
	Chitin and N-acetylglucosamine utilization	Chitinase (EC 3.2.1.14)	1468, 2556, 2772, 3118, 4199, 5580
		Kinase similar to eukaryotic-like N-acetylglucosamine kinase	3386
	Chitin and N-acetylglucosamine utilization, Sialic Acid Metabolis	N-acetylglucosamine-6-phosphate deacetylase (EC 3.5.1.25)	152, 1149, 1401, 2191
	Chitin and N-acetylglucosamine utilization	Glucosamine-6-phosphate deaminase (EC 3.5.99.6)	151, 1400, 2190
	Chitin and N-acetylglucosamine utilization	Beta-hexosaminidase (EC 3.2.1.52)	304, 1419, 1950, 3645, 4260, 4717
	Methionine degradation	Methionine aminopeptidase (EC 3.4.11.18)	1641, 1736
		S-adenosylmethionine synthetase (EC 2.5.1.6)	1715
		S-adenosylmethionine decarboxylase proenzyme (EC 4.1.1.50)	517
		Spermidine synthase (EC 2.5.1.16)	508

ABC transporters

ABC Transporter oligopeptide (TC 3.A.1.5.1)	Oligopeptide transport system permease protein OppC	33, 439, 1303
ABC Transporter oligopeptide (TC 3.A.1.5.1)	Oligopeptide transport system permease protein OppB	34, 440, 1304
ABC Transporter oligopeptide (TC 3.A.1.5.1)	Oligopeptide transport system permease protein OppF	31, 437, 846, 1301
ABC Transporter oligopeptide (TC 3.A.1.5.1)	Oligopeptide transport system permease protein OppD	32, 438, 658, 659, 702, 847, 1302
ABC Transporter oligopeptide (TC 3.A.1.5.1)	Oligopeptide ABC transporter, periplasmic oligopeptide binding protein OppA	35, 441, 850, 1305, 4252
ABC Transporter oligopeptide (TC 3.A.1.5.1)	ABC Transporter ATP-binding protein	443
ABC Transporter branched-chain amino acid (TC 3.A.1.4.1)	High-affinity branched-chain amino acid transport system permease protein LivH	1605
ABC Transporter branched-chain amino acid (TC 3.A.1.4.1)	Branched-chain amino acid transport ATP-binding protein LivF	1602
ABC Transporter branched-chain amino acid (TC 3.A.1.4.1)	Branched-chain amino acid transport system permease protein LivM	420, 1604
ABC Transporter branched-chain amino acid (TC 3.A.1.4.1)	Branched-chain amino acid ABC transporter, amino acid binding protein	1606
ABC Transporter branched-chain amino acid (TC 3.A.1.4.1)	Branched-chain amino acid transport ATP binding protein LivG	1603
ABC Transporter dipeptide (TC 3.A.1.5.2)	Dipeptide transport system permease protein DppB	661, 704, 849
ABC Transporter dipeptide (TC 3.A.1.5.2)	Dipeptide transport system permease protein DppF	657
ABC Transporter dipeptide (TC 3.A.1.5.2)	Dipeptide transport system permease protein DppC	703, 848
Putative Polar amino acid transporter	Polar amino acid transport system substrate binding protein	2739,3237, 5056

Putative Polar amino acid transporter	Polar amino acid transport system permease protein	2740, 3238, 5055
Putative Polar amino acid transporter	Polar amino acid transport system ATP-binding protein (EC 3.6.3.21)	2741, 3239, 5054
Putative sugar ABC transporter (ytf cluster)	Putative sugar ABC transport system, periplasmic binding protein YtfQ precursor	4161, 2002, 1995, 4161
Putative sugar ABC transporter (ytf cluster)	utative sugar ABC transport system, permease protein YtfF	1999
Putative sugar ABC transporter (ytf cluster)	Putative sugar ABC transport system, ATP-binding protein YtfR (EC 3.6.	1997
Putative sugar ABC transporter (ytf cluster)	Permease protein (YtfT)	1998
	Permease protein (YtfF)	1999
D-ribose utilization	Ribose ABC transport system, ATP-binding protein RbsA (TC 3.A.1.2.1)	421, 464, 1991, 2838, 4089, 4154, 4151, 4291, 3286, 1429, 455, 597, 411, 2838, 3286, 4089, 4154, 4291
D-ribose utilization	Ribose ABC transport system, permease protein RbsC (TC 3.A.1.2.1)	455, 1990, 4088, 4155, 4289, 4290, 3287, 1428, 5094, 3428, 596, 409, 410, 3287, 3428, 4088, 4155, 4289, 4290, 5094
D-ribose utilization	Ribose ABC transport system, high affinity permease RbsD (TC 3.A.1.2.1)	1430
D-ribose utilization	Ribose ABC transport system, periplasmic ribose-binding protein RbsB (TC 3.A.1.2.1)	1427, 403, 2110, 595, 408, 413
D-ribose utilization	Ribose/xylose/arabinose/galactoside ABC-type transport systems, permease componer	1989
D-ribose utilization	Ribose/xylose/arabinose/galactoside ABC-type transport systems, periplasmic sugar bi	1993
Xylose utilization	Xylose ABC transporter, substrate-binding component	3285, 1423, 3429, 1752, 2825, 3285, 3429, 3641
Xylose utilization	Xylose ABC transporter, periplasmic xylose-binding protein XylF	2106, 2107
Xylose utilization	D-xylose transport ATP-binding protein XylG	2105
Xylose utilization	Xylose ABC transporter, permease component	683, 1741, 2443, 2572, 2826, 2906, 3403, 3404, 3642, 4345, 4576, 4726, 5219
Xylose utilization	Sugar ABC transporter permease protein ycjP	682
Xylose utilization	ABC transporter, substrate-binding protein	681
Xylose utilization	Probable ABC transporter permease protein ytcP	1740, 4344
Xylose utilization	Possible alpha-xyloside ABC transporter, permease component	3213
Inositol catabolism	Inositol transport system sugar-binding protein	401
Inositol catabolism	Inositol transport system ATP-binding protein	3430
	Inositol transport system permease protein	399
Fructooligosaccharides(FOS) and Raffinose Utilization	Multiple sugar ABC transporter, substrate-binding protein	1090, 1617, 4423
	Multiple sugar ABC transporter, membrane-spanning permease protein MsmG	1091
	Multiple sugar ABC transporter, membrane-spanning permease protein MsmF	1092, 1618
Maltose and Maltodextrin Utilization	Maltose/maltodextrin ABC transporter, permease protein MalG	1619, 1979, 2189, 2400, 3689, 3966, 4941, 5289
Maltose and Maltodextrin Utilization	Maltose/maltodextrin ABC transporter, permease protein MalF	1980, 2188, 2399, 2648, 3688, 3965, 4942
Maltose and Maltodextrin Utilization	Maltose/maltodextrin ABC transporter, substrate binding periplasmic protein MalE	1981, 2028, 2397, 2854, 3964, 4943, 5335
Maltose and Maltodextrin Utilization	maltose/maltodextrin transport permease	2444, 2573, 4575
	Polysaccharide ABC transporter substrate-binding protein	2445
Fructooligosaccharides(FOS) and Raffinose Utilization, Maltose a	Multiple sugar ABC transporter, ATP-binding protein	2881
Glycerol and Glycerol-3-phosphate Uptake and Utilization	Glycerol-3-phosphate ABC transporter, ATP-binding protein UgpC (TC 3.A.1.1.3)	2401, 3690
L-Arabinose utilization	L-arabinose transport system permease protein (TC 3.A.1.2.2)	2104
Lactose and Galactose Uptake and Utilization	Galactose/methyl galactoside ABC transport system, ATP-binding protein MglA (EC 3.6	3550, 400, 456
Lactose and Galactose Uptake and Utilization	D-galactose-binding periplasmic protein MglB (TC 3.A.1.2.3)	3549
Lactose and Galactose Uptake and Utilization	Galactose/methyl galactoside ABC transport system, permease protein MglC (TC 3.A.1.	3551
Bacterial Chemotaxis, Lactose and Galactose Uptake and Utilizat	Galactose/methyl galactoside ABC transport system, D-galactose-binding periplasmic p	3549
Lactose and Galactose Uptake and Utilization	Galactose/methyl galactoside ABC transport system, ATP-binding protein MglA (EC 3.6	3550
Lactose and Galactose Uptake and Utilization	Galactose/methyl galactoside ABC transport system, permease protein MglC (TC 3.A.1.	3551

ABC type sugar transport systems	Permease component	1422
	Permease	1421
	Periplasmic component	5093
Various polyols ABC trsnsporter	ATP binding component	5095
Fructose utilization	Fructose ABC transporter, substrate-binding component FrcB	454
Nucleoside ABC transporter	periplasmic nucleoside-binding protein	2837, 2013, 2143
	Permease protein 1	2839, 2015, 2145
	Permease protein 2	2840, 2016, 2146
ABC transporter	ATP-binding protein	425, 2014
ABC sugar transporter	periplasmic binding protein	4295
Unspecified monosaccharide ABC transport system	ATP- binding protein	2144
Fructooligosaccharides(FOS) and Raffinose Utilization	Multiple sugar ABC transporter, membrane-spanning permease protein MsmG	159, 1365, 2078, 2182, 2471, 2766, 3253, 3392, 3455, 3838, 4420, 4631, 4639, 4835, 5081
Fructooligosaccharides(FOS) and Raffinose Utilization	Multiple sugar ABC transporter, membrane-spanning permease protein MsmF	160, 1366, 2181, 2470, 2488, 2765, 3454, 3837, 4632, 4640, 4836, 5082
Fructooligosaccharides(FOS) and Raffinose Utilization	Multiple sugar ABC transporter, substrate-binding protein	161, 1364, 2076, 2473, 2491, 2764, 3453, 3481, 3836, 4630, 4638, 4834, 5079
Fructooligosaccharides(FOS) and Raffinose Utilization	Multiple sugar ABC transporter, ATP-binding protein	2881, 3691
Chitin and N-acetylglucosamine utilization	N-Acetyl-D-glucosamine ABC transport system, permease protein 2	559, 1463, 1513, 1585, 1957, 2031, 2090, 2267, 2749, 3149, 3269, 4407, 5187
Chitin and N-acetylglucosamine utilization	N-Acetyl-D-glucosamine ABC transport system, permease protein 1	561, 1013, 1464, 1514, 1582, 1586, 1958, 2032, 2077, 2091, 2269, 2748, 3149, 3628, 3608, 4409, 4421, 5186, 5288, 5321, 5336
Chitin and N-acetylglucosamine utilization	ABC transport system, sugar-binding protein	562, 1014, 1465, 1515, 1587, 1959, 2013, 2092, 2180, 2270, 2740, 3147, 3267, 3511, 4411, 5185
	Putative sugar ABC transporter	1012
Chitin and N-acetylglucosamine utilization	Predicted chitobiose ABC transport system II, sugar-binding protein	2647
Chitin and N-acetylglucosamine utilization	N-Acetyl-D-glucosamine ABC transport system, permease protein	3507
Glycerol and Glycerol-3-phosphate Uptake and Utilization	Glycerol-3-phosphate ABC transporter, periplasmic glycerol-3-phosphate-binding protei	2187, 3610
Glycerol and Glycerol-3-phosphate Uptake and Utilization	Probable glycerol transport protein	2855
No category	ABC-type multidrug transport system, ATPase component	2492
No category	ABC-type transport system involved in multi-copper enzyme maturation, permease con	2493
No category	ATP-binding protein of ABC transporter	2549, 2551
No category	ABC-type polysaccharide transport system, permease component	726, 2552, 3212, 3253, 3508
No category	ABC transporter, permease protein	2490, 2553, 2907, 3393, 4703
No category	ABC transporter, substrate-binding protein	723, 2554, 2908, 3211, 3391, 3405, 4577, 4724
No category	ABC transporter, membrane spanning protein	2649
No category	sugar ABC transporter permease	1421, 2827, 3643
No category	Nucleoside ABC transporter, periplasmic nucleoside-binding protein	2837
No category	Nucleoside ABC transporter, permease protein 1	2839
No category	Nucleoside ABC transporter, permease protein 2	2840
No category	Glycine betaine ABC transport system, glycine betaine-binding protein OpuAC	2861
No category	ABC transporter permease component	3609
No category	Predicted erythritol ABC transporter 1, substrate-binding component	3687
No category	Sugar ABC transporter	4090
No category	ABC transporter, ATP-binding protein	4151, 4702, 4705
No category	ABC sugar transporter, periplasmic binding protein	4295
No category	Probable ABC transporter permease protein ytcP	4725, 5220
No category	similar to ABC-type sugar transport system periplasmic component	5093
No category	Various polyols ABC transporter, ATP-binding component	5095
No category	ABC transport system, sugar-binding protein	5320
No category	ABC-type sugar transport system, permease component	5337
No category	Possible fucose ABC transporter, permease component	463

No category	Possible fucose ABC transporter, substrate-binding component	461, 465
No category	putative sugar ABC transporter permease	725
Inositol catabolism	5-keto-2-deoxygluconokinase (EC 2.7.1.92)	1000
Inositol catabolism	Inositol transport system, Permease protein	1001, 399
Inositol catabolism	Inositol transport system, ATP-binding protein	1002, 3430
Inositol catabolism	Inositol transport system, Sugar binding protein	1003, 401
Inositol catabolism	Epi-inositol hydrolase (3.7.1.-)	1004
Inositol catabolism	Inosose dehydratase (EC 4.2.1.44)	1005, 1007
Inositol catabolism	5-deoxyglururonate isomerase (EC 5.3.1.30)	1006
Inositol catabolism	Myo-Inositol dehydrogenase (EC 1.1.1.18)	1008, 397
	Inosose isomerase (EC 5.3.99.-)	398
L-Arabinose utilization	Ribulokinase (EC 2.7.1.16)	
L-Arabinose utilization	L-arabinose transport system permease protein (TC 3.A.1.2.2)	
L-Arabinose utilization	L-arabinose isomerase (EC 5.3.1.4)	
L-Arabinose utilization	Transcriptional repressor of arabinoside utilization operon, GntR family	
L-Arabinose utilization	Alpha-L-arabinofuranosidase II precursor (EC 3.2.1.55)	

Proteases and peptidases

	alkaline serine protease	3934
	Transglutaminase-like enzyme, putative cysteine protease	4118
A cluster relating to Tryptophanyl-tRNA synthetase	FIG004556: membrane metalloprotease	4119
	Protease (EC 3.4.-.-)	1113, 1122, 4215
DNA repair, bacterial	SOS-response repressor and protease LexA (EC 3.4.21.88)	4276
	Secreted protease metal-dependent protease	4309
Phosphoglycerate mutase protein family	Carboxyl-terminal protease (EC 3.4.21.102)	145, 1750, 2886
Small acid-soluble spore proteins	Endopeptidase spore protease Gpr (EC 3.4.24.78)	289
	Spore protease GPR related protein	2244
Proteolysis in bacteria, ATP-dependent	ATP-dependent protease La (EC 3.4.21.53) Type I	1295
Proteolysis in bacteria, ATP-dependent	ATP-dependent Clp protease ATP-binding subunit ClpX	1296, 5504
Proteolysis in bacteria, ATP-dependent, cAMP signaling in bacter	ATP-dependent Clp protease proteolytic subunit (EC 3.4.21.92)	1297
Proteolysis in bacteria, ATP-dependent	ATP-dependent Clp protease, ATP-binding subunit ClpC / Negative regulator of genetic	3573
	Putative stomatin/prohibitin-family membrane protease subunit PA4582	1461
	Putative stomatin/prohibitin-family membrane protease subunit YbbK	1561
	Putative activity regulator of membrane protease YbbK	1562
	FIG056164: rhomboid family serine protease	1912
	CAAX amino terminal protease family protein	2061, 2293
	Alpha-lytic protease precursor (EC 3.4.21.12) (Alpha-lytic endopeptidase)	2310
SA:14-24	Serine protease, DegP/HtrA, do-like (EC 3.4.21.-)	2503
Proteolysis in bacteria, ATP-dependent	ATP-dependent protease La (EC 3.4.21.53) Type II	2936
	Protease precursor	3739
	Membrane-associated zinc metalloprotease	4454
	Hydrogenase maturation protease delta subunit, HyaD-like	4596
	putative metal-dependent membrane protease	4884

Proteolysis in bacteria, ATP-dependent, Ribosome recycling relat	ATP-dependent Clp protease ATP-binding subunit ClpA	5051
	Cell wall-associated protease precursor (EC 3.4.21.-)	5469
CBSS-84588.1.peg.1247, Metallocarboxypeptidases (EC 3.4.17.-),	D-alanyl-D-alanine carboxypeptidase (EC 3.4.16.4)	41, 4110, 4122, 4223,
	Peptidase, M20/M25/M40 family	147
Protein degradation	Aminopeptidase YpdF (MP-, MA-, MS-, AP-, NP- specific)	224
	peptidase, U32 family large subunit [C1]	266
	Oligoendopeptidase F (EC 3.4.24.-)	590
Aminopeptidases (EC 3.4.11.-)	Cytosol aminopeptidase PepA (EC 3.4.11.1)	110
CBSS-176299.4.peg.1292, Signal peptidase	Signal peptidase I (EC 3.4.21.89)	909
	Aspartyl aminopeptidase (EC 3.4.11.21)	924
Peptidoglycan Biosynthesis	Multimodular transpeptidase-transglycosylase (EC 2.4.1.129) (EC 3.4.-.-)	1030, 1031
Muramoyltetrapeptide carboxypeptidase (EC 3.4.17.13)	Muramoyltetrapeptide carboxypeptidase (EC 3.4.17.13)	1219
Metalloendopeptidases (EC 3.4.24.-)	Microbial collagenase (EC 3.4.24.3)	1348
Aminopeptidases (EC 3.4.11.-)	Xaa-Pro aminopeptidase (EC 3.4.11.9)	1393
CBSS-312309.3.peg.1965, Translation termination factors bacteri	Methionine aminopeptidase (EC 3.4.11.18)	1641, 1736
CBSS-393121.3.peg.2760	Membrane proteins related to metalloendopeptidases	711, 1724, 2049, 2501, 2885, 3460, 5414, 5600, 5613
CBSS-176299.4.peg.1292, Signal peptidase	Signal peptidase I (EC 3.4.21.89)	1775, 4012
CBSS-393121.3.peg.2760	Peptidase, M23/M37 family	1861, 1886
	Aminopeptidase Y (EC 3.4.11.-)	2095
Heat shock dnaK gene cluster extended	Signal peptidase-like protein	2152
	Oligoendopeptidase F (EC 3.4.24.-)	2250
	Alpha-lytic protease precursor (EC 3.4.21.12) (Alpha-lytic endopeptidase)	2310
	aminopeptidase	2338
	Deblocking aminopeptidase (EC 3.4.11.-)	2342
	Peptidase, S41 family	2714, 3462
	Oligoendopeptidase F (EC 3.4.24.-)	3578
CBSS-84588.1.peg.1247, Metallocarboxypeptidases (EC 3.4.17.-),	D-alanyl-D-alanine carboxypeptidase (EC 3.4.16.4)	3101
	Leader peptidase (Prepilin peptidase) (EC 3.4.23.43) / N-methyltransferase (EC 2.1.1.-)	3189
Peptidoglycan Biosynthesis	Multimodular transpeptidase-transglycosylase (EC 2.4.1.129) (EC 3.4.-.-)	3649
	Tripeptide aminopeptidase (EC 3.4.11.4)	3650
	Zn-dependent peptidase, insulinase family	3854
	probable metal-dependent peptidase	3977
CBSS-1806.1.peg.3045, CBSS-350688.3.peg.1509	FIG007959: peptidase, M16 family	4042
Metallocarboxypeptidases (EC 3.4.17.-), Murein Hydrolases, Recy	Muramoyltetrapeptide carboxypeptidase (EC 3.4.17.13)	4055
	similar to sp:LSPA_STACA LIPOPROTEIN SIGNAL PEPTIDASE (EC 3.4.23.36) (PROLIPOPR	4174
Metallocarboxypeptidases (EC 3.4.17.-)	Thermostable carboxypeptidase 1 (EC 3.4.17.19)	4200
Dipeptidases (EC 3.4.13.-), Recycling of Peptidoglycan Amino Aci	Aminoacyl-histidine dipeptidase (Peptidase D) (EC 3.4.13.3)	4258
Lipoprotein Biosynthesis, Signal peptidase	Lipoprotein signal peptidase (EC 3.4.23.36)	4333, 4892
	Gamma-D-glutamyl-L-diamino acid endopeptidase I (EC 3.4.19.11)	4352
	Cell wall endopeptidase, family M23/M37	4449
Sporulation gene orphans	Sporulation sigma-E factor processing peptidase (SpoIIGA)	4514

Hydrogenases

G3E family of P-loop GTPases (metallocenter biosynthesis), NiFe	[NiFe] hydrogenase metallocenter assembly protein HypE	4591
G3E family of P-loop GTPases (metallocenter biosynthesis), NiFe	[NiFe] hydrogenase metallocenter assembly protein HypD	4592
G3E family of P-loop GTPases (metallocenter biosynthesis), NiFe	[NiFe] hydrogenase metallocenter assembly protein HypC	4593
G3E family of P-loop GTPases (metallocenter biosynthesis), NiFe	[NiFe] hydrogenase metallocenter assembly protein HypF	4594
G3E family of P-loop GTPases (metallocenter biosynthesis), NiFe	[NiFe] hydrogenase nickel incorporation-associated protein HypB	2579
G3E family of P-loop GTPases (metallocenter biosynthesis), NiFe	[NiFe] hydrogenase nickel incorporation protein HypA	2581
Hydrogenases	Hydrogenase maturation protease delta subunit, HyaD-like	4596
Hydrogenases	[Ni/Fe] hydrogenase, group 1, large subunit	4597, 4599
Hydrogenases	[FeFe]-hydrogenase maturation protein HydE	138

Hydrogenases	[FeFe]-hydrogenase maturation protein HydF	4381
Hydrogenases	Periplasmic [Fe] hydrogenase large subunit (EC 1.12.7.2)	95,2068, 2901, 3896
Hydrogenases	Periplasmic [Fe] hydrogenase (EC 1.12.7.2)	96, 98, 4378
Hydrogenases	NAD-reducing hydrogenase subunit HoxE (EC 1.12.1.2)	2066, 2898, 3894
Hydrogenases	NAD-reducing hydrogenase subunit HoxF (EC 1.12.1.2)	2067, 2900, 3895
Hydrogenases	NADP-reducing hydrogenase, subunit B	2899

**Flagella**		
	RNA polymerase sigma factor for flagellar operon	4466
Flagellar motility	Signal transduction histidine kinase CheA (EC 2.7.3.-)	4471, 1402
Flagellar motility	Flagellar synthesis regulator FleN	4474
Flagellar motility	Flagellar biosynthesis protein FlhF	4475
Flagellar motility	Flagellar biosynthesis protein FlhA	4476
Flagellar motility	Flagellar biosynthesis protein FlhB	4477, 4017
Flagellar motility	Flagellar biosynthesis protein FliR	4478
Flagellar motility	Chemotaxis regulator - transmits chemoreceptor signals to flagelllar motor components CheY	4482, 4418, 279
Flagellar motility	Flagellar motor switch protein FliN	4483
Flagellar motility	Flagellar motor switch protein FliM	4484
Flagellar motility	Flagellar motor rotation protein MotA	4487
Flagellar motility	Flagellar basal-body rod modification protein FlgD	4491
Flagellar motility	Flagellum-specific ATP synthase FliI	4495
Flagellar motility	Flagellin	1684

Polyketid biosynthetic cluster		
	Start (gene number in RAST)	End (gene number in RAST)
1	4638	4674
2	4868	4879
3	4883	4940
4	328	363
5	1095	1192
6	1389	1409
7	1504	1536
8	2596	2652
9	3299	3348
