# Supplementary figures and images for: Genome Analysis of Vallitalea guaymasensis Strain L81 Isolated from a Deep-Sea Hydrothermal Vent System
Source: Microorganisms. 2018 Jul 4;6(3):63. doi: 10.3390/microorganisms6030063 (PMC6163223; doi:10.3390/microorganisms6030063)

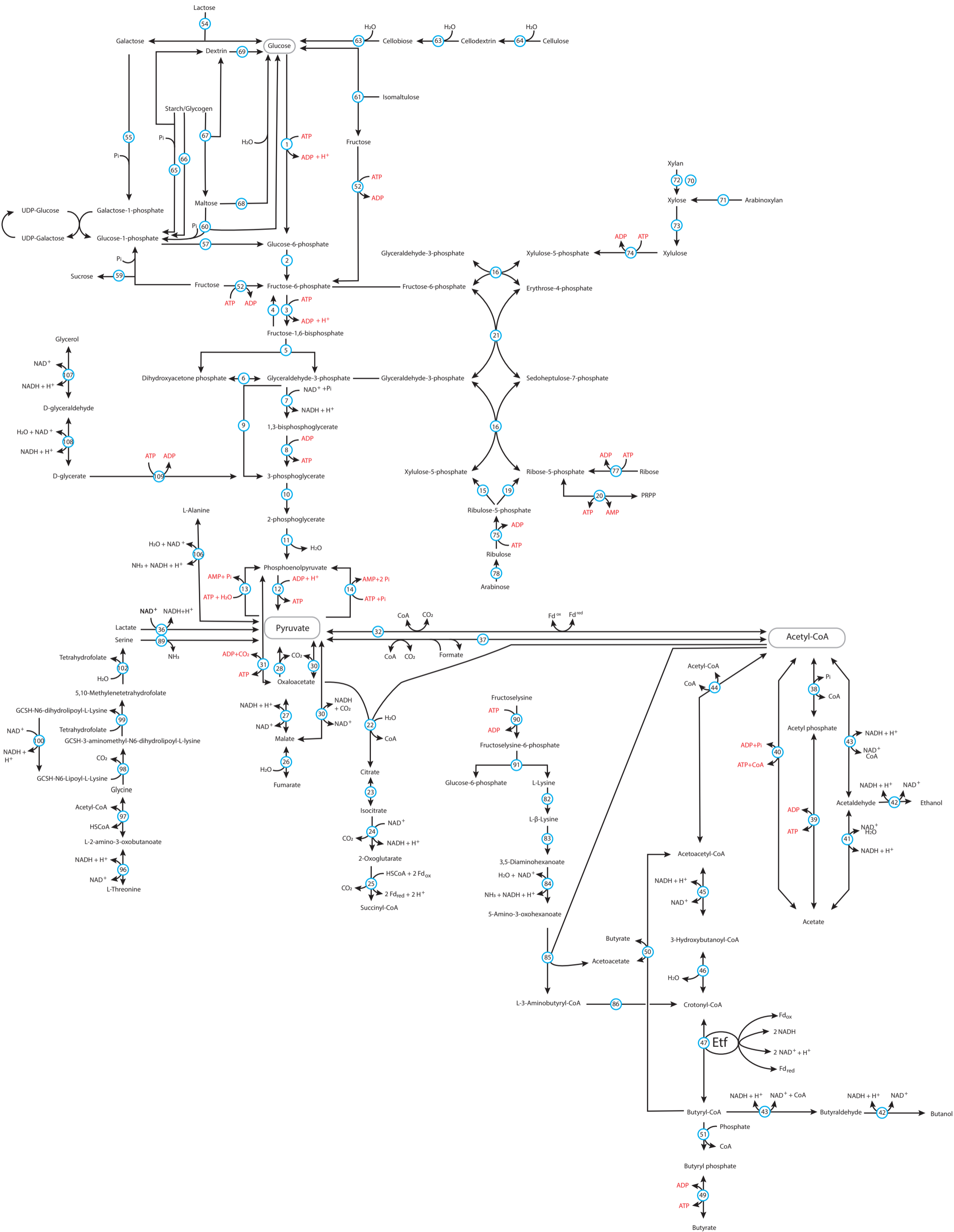

Supplement: Supplementary file 1 [file microorganisms-06-00063-s001.zip › Core metabolism_color2.pdf]
